# Germinal Matter

**Published:** 1870-03

**Authors:** Thruston Wolfe


### ARTICLE III.

### *Germinal Matter.*

By Thruston Wolfe, D.D.S.

Life may be considered as the production of a physiological condition of an organization. It may be divided into two essential states or conditions. First, the material condition, and second, the dynamical. The material condition consists of food, oxygen and water, each being directly essential to the maintenance of a normal action of the organs of the body. The dynamical condition consists of light, heat and electricity. They also bear an important relation in the great laboratory of life. These two conditions work in such harmony as to bring to bear such results as achieve the hystogenetic and histolytic processes.

In the consumation of these results there are found to be two distinct forces at work, viz: First, vegetative force and second, animal force. The first has the power of assimilation, propagation and development .The second power exhibits the development of muscular, nerve and brain forces. There must be a basis upon which these forces may act, this we call "Germinal Matter." This being the case, we naturally enquire what this germinal matter is? The answer is, it is the great constructive basis substance of organic life. It is that from which all the tissues are formed.

In the fœtus there are noticed at first a number of cells which have the power of assimilation and propagation. These cells multiply very rapidly in a mass and after a while they begin to form themselves with a certain definite outline showing somewhat the shape of the body to be formed. These cells develope into tissue in a manner hereafter to be described. Germinal matter asserts its power in both vegetable and animal kingdoms, but is decidedly more persistent in the vegetable than the animal kingdom. To prove this we will cite a case of some wheat which was found in a pyramid supposed to have been there at least two thousand years, and after being procured and planted was found to germinate, grow and bring forth species after its kind. So also even some raspberry seeds found in the stomach of a man who had been petrified, who had on his person some coins of the date and reign of Emperor Hadrian. No definite idea of the length of time that elapsed from the time of eating till they were found. But certainly a great many years. These seeds on being experimented with were found to germinate, grow and bear fruit of the same species. These and other instances show that notwithstanding the long periods that elapsed between the time of their first growth and perfection to that of the second, the germinal matter of these seeds did not lose its power of assimilation, propagation and development.

But germinal matter acts in a different manner in the animal kingdom. It is found in a clear, limpid fluid called pabulum and has a nucleus around which it forms, grows and multiplies, each little mass dividing and budding forming more masses as large as was the parent mass. It is manufactured of this pabulum in which it is found, and is rarely more than .0001 of an inch in diameter, each little mass having an approximate size. Should it grow larger than its normal size, it could not act in a histogenetic manner. For the nutriment it supplies cannot be derived from the centre of the mass, it being to thick and large for the proper current to enter and carry the nourishment to the parts required. Therefore it must lie useless and its first is always in a retrograding manner. It has been remarked that germinal matter has three stages of existence, viz: First, assimilation, second, propagation, and third, development.

Assimilation is that process by which the germinal matter forms to itself the material in which it is placed, making itself instead of leaving it pabulum.

Propagation is that process in which the germinal matter multiplies itself and instead of remaining one mass, becomes many. This process is done by dividing and budding. The budding is distinguished from the other process by a small speck seen on the side of the mass, and growing forms a mass as large as the parent and is nipped off. The other process is the little masses simply dividing and becoming other masses. The developmental power is distinguished from the other two in that the germinal matter has run its course and accomplished its aim and has formed tissues, organs. The exhibition of these three powers is entirely requisite for the formation of healthy tissues. These powers are respectively denominated the first, second and third powers.

They do not always exhibit their inherent right in their attempt at development. For when there is an abnormal arrangement in the germinal matter at work, the developmental process is retarded and often completely arrested. The failure of the exhibition of either the first or second powers, always arrests the third. After it has exhibited the third or developmental power, it is formed material; this formed material being the result of a normal action of the vegetative forces. There is a test showing a distinctive feature between germinal matter and formed material. This test Beale calls the "carmine test," it is composed of ammonia, carmine and glycerine.

When an application of this carmine test is made to germinal matter, it turns it red, whereas it does not change the formed material at all. "On close examination the muscular fibres are found to consist of minute particles strung together like beads," and between these are found the nutrient masses which supply the tissues as fast as they are wasted during the continual changes that are going on during life.

Special tissues are formed by a special power inherent in the germinal matter of those tissues. Germinal matter is found wherever tissues as to be formed. The three vegetative powers are very highly demonstrated in the formation of epidermic scales and epitheloid cells. The epidermic scales are found on the skin and the epitheloid cells in the mucous membrane. The scales are formed from the germinal matter which being first in the mucus immediately external to the aveola tissue and exhibiting the three powers, finally terminating upon the periphery and by lateral pressure become flattened branny scales. The epithelial cells are also formed from the mucus globules as first noticed in the pabulum and displaying the three vegetative forces.

The function of the skin is for the external covering and protection of the body and osmotic action. That of the mucus membrane is for the internal lining of the body and osmotic action.

Germinal matter asserts its inherent right when in a normal condition under all circumstances. For instance, in the process of grafting and budding, the grafts or buds grow and bear fruit or flowers after its original kind. So also if the germinal of the periosteum of bone be removed to another part of the body and planted in the soft tissues it will give birth to a bony formation and not the kind in which it is planted. In the formation of bone we notice at first a cartilaginous mass composed of cells placed promiscously in it. This stage is called the simple cellular, afterwards, however, these cells begin to arrange themselves in lines which are to become in the bone the Haversian canals and the medullary channel. These Havesian canals run into each other, and divide sometime, and give an irregular power condition to the bone. Radiating from the Haversian canals are a number of small tubes called canaliculi. A great number of laminæ are found around the Haversian canals and are very thin layers in close proximity with each other. These laminæ enter largely into the formation of the bone.

Along the line of these canaliculi are seen little cavities which have leading from them other canaliculi, which also run into other little spaces and then into the Havernian canals again. These little spaces or cavities are termed Lacuna spaces and contain lacuna masses which is the germinal matter of bone. The canaliculi are the plasmatic tubes. The medullary canal is formed by the absorption of the parts along the row or line of cells which were formed in that portion of the bone which is to contain it Ossification of these canal and cell walls gives the bony substances. Bone it composed of organic or animal matter, and inorganic or earthy matter. The organic composes about one-third o the bone ; it is gelatine and bloodvessels. The earthy matter constitutes two-thirds ; it is composed of carbonate and phosphate of lime, fluoride of calcium, phosphate of magnesia, soda and chloride of sodium. This bony substance forms the greater part of the hard tissues of the body and gives a framework for the attachment of the soft tissues of the body and protection to more vital parts, such as the brain, spinal cord, heart etc. It is termed the skeleton. There are 204 bones in the body and are called long, flat and irregular. They grow in circumference from the germinal matter supplied by the periosteum on its external surface, and from nutrition supplied by the medullary. They grow in length by another process. Near either extremity in the young bone is found a division of the parts and connected by a layer of cartilage. From this cartilage is supplied an amount of germinal matter which forms new bone, and as the cartilage next the free surface of the bone becomes ossified new is formed. Thus the process of ossification and reproduction is going on regularly till the bones have reached their maximum length, at which period the whole of the said cartilage becomes ossified; when this occurs the bone can never grow longer.

The teeth are formed in somewhat a different manner, at about the sixth week of intra uterine life there are noticed two folds of mucous membrane in the place where the upper or lower jaw bones are to be formed. The outer of these folds is for the cheek and the inner for the tongue. About the same week a ridge begins between these folds running from behind forward to meet in the median line. This ridge divides the groove into two grooves. About the seventh week after conception is seen the germ of first temporary molar in the primitive dental groove of the upper jaw, in the form of a "simple free granular papilla," of an ovoidal shape.

One after another of these papillæ are formed in the primitive dental groove, and at the same time processes are sent out from the outer and inner walls of the groove, forming follicles for the papillæ. This is denominated the follicular stage. The mouth of the follicles become more developed and grow up and close in the papillæ. The lids covering these papillæ are termed opercula. This is called the saccular stage. After the close of the opercula, there is a cresent shaped space left immediately behind the minor opercula which recedes toward the root or base of the tooth germ, and passing finally beneath it or nearly so. This cavity is the cavity of reserve, and in it is to be found the permanent teeth. It has in its bottom a papillæ which gives rise to the permanent tooth. After the closing in of the opercula over the papillæ, it becomes intensely vascular as well as the inner portion of the dental sac. It is not until then that the germinal matter is formed in the papilla that forms the tooth. The papilla may now be called the pulp.

The external surface of the pulp throws off nutrient fluid or germinal matter simultaneously with that of the dental sac, and they then begin the formation of the enamel and dentine. The dentine is formed from without inward from the junction of the enamel and dentine, by the germinal matter from the pulp; calcification begins and encroaches upon the pulp, and is composed of fibrillæ, some of which do not become calcified at the time of the formation of the dentine. These fleshy fibrillæ are found to be an organic continuation with the pulp. They give sensitiveness to the dentine and are liable at any time to calcify. In young persons these fibrillæ are more numerous and larger than in older ones. The pulp in the teeth of young persons is much larger than old ones also, for as we advance in age, the external portion of the pulp calcifies and recedes more toward the root or apex of the teeth, hence the greater liability of toothache in young persons. The enamel is formed from the coronal extremity of the dentine outwards. The germinal matter from the internal portion of the dental sace, begins to form the enamel on the line of junction with the dentine. It is formed in solid hexagonal prisms placed so close together as to form a solid mass. These prisms or rods sit with their ends pointing outward, so that the force of grinding and wearing will be directly across their ends and not along the line of their bodies. The calcification is thorough in the enamel and no change save that of disintegration can take place. The cementum or crusta petrosa is formed by the germinal matter given by the dental sac in that portion of the tooth. Its structure is analagous to bone. It occupies that portion of the tooth which is external to the dentine, below the neck of the tooth, and is less liable to disease than any other portion of the tooth; and the disease is generally different.

Germinal matter does not always exhibit a healthy action. Very frequently it displays abnormal conditions which are irreparable within itself.

"The law of low vitality is arrest of development." Thus when germinal matter makes an abortive attempt at development, we know at once that its vitality is lowered. In the formation of pus, there is an arrest of the third or developmental power, an inordinate exhibition of the first and second, or the powers of assimilation and propagation. The vitality of the masses of germinal matter is lost in proportion as the work of propagation progresses. For when the vitality is gone, we notice a greasy, creamy, thick and yellowish mass which has become entirely passive in its action. Pus reaches the free surface by three methods: First by passing directly through the epithelial cells by osmodic action and appearing on the surface. Second, by softening the cells and causing them to give away and appearing on the surface. Third, by bursting the tissues by tension and coming through by force and appearing on the periphery. Mucus, mucine, fibrine and the amyloid change are all exhibitions of the developmental power partially arrested. The hard tissues are also liable to disease and undergo a hystoitic process as well as the soft tissues. In caries and necrosis of bone, for instance there is a decided and complete arrest of the three powers of histogenesis. Caries of bone is that condition in which is found the animal matter in an abnormal condition, the lacuna masses not performing healthy functions and a suppurative process takes place; caries is the first step to necrosis. In it is found the first and second powers exhibited and an abeyance of the third. It ulcerates and discharges a fetid matter like the soft tissues.

Necrosis is complete and absolute death of bone. That portion of the bone has lost all its vitality. The germinal matter having lost the force of all its powers.

The teeth are also attacked by caries, but there is a marked difference between the caries of teeth and bone. In teeth it consists of a decomposition of the inorganic basis and the disorganization of the animal framework, and never throw out fungus granulations as bone does. Caries may be classed into three varieties, viz: Chemical, Vital and Chemico Vital.

The chemical cause of caries is the direct action upon the teeth by such agents as destroy its organization. The vital is the death of the animal framework of the organ and then acted upon by corrosive agents.

The chemico vital is the chemical action first and after the death of the animal matter of the tooth.

The causes of caries are various. Acids of the mouth, the use of strong medicines and salivary calculus upon them, the condition or state of the health, irregularity, and in fact everything that does not accord with the normal condition of them will produce it. They will never undergo a reparative process as bone does. Nature can never bring about a histogenesis.

